# Risk stratification of postoperative enteral feeding intolerance using explainable machine learning in oral cancer free flap reconstruction

**DOI:** 10.3389/fnut.2026.1815516

**Published:** 2026-06-04

**Authors:** Baolin Jia, Yao Wen, Bo Deng, Gaoyan He, Jun Ren, Guixin Li, Xianjie Zheng, Xiaojuan Wu, Sen Yang

**Affiliations:** 1 Department of Oral and Maxillofacial Surgery, Suining Central Hospital, Suining, Sichuan Province, China; 2 Department of Gastroenterology, Suining Central Hospital, Suining, Sichuan Province, China; 3 Department of General Practice, Suining Central Hospital, Suining, Sichuan Province, China; 4 Department of Respiratory Medicine and Critical Care Medicine, Suining Central Hospital, Suining, Sichuan Province, China

**Keywords:** enteral nutrition, feeding intolerance, free flap reconstruction, machine learning, oral cancer

## Abstract

**Background:**

Enteral nutrition (EN) is essential after free flap reconstruction for oral cancer; however, feeding intolerance (FI) frequently limits adequate nutritional delivery. Existing prediction tools are primarily derived from general intensive care unit populations and may not adequately reflect the unique metabolic and inflammatory vulnerabilities of this surgical cohort. We aimed to develop and internally validate an interpretable machine learning model for the early prediction of postoperative FI.

**Methods:**

In this single-center retrospective study, 752 patients undergoing radical resection with free flap reconstruction (between 2017 and 2025) were included. FI was defined according to established consensus criteria. A total of 35 perioperative variables were evaluated. Feature selection was performed using least absolute shrinkage and selection operator (LASSO) regression. Seven machine learning algorithms were trained and validated on an independent hold-out set. Model performance was assessed by the area under the receiver operating characteristic curve (AUC), calibration, and decision curve analysis. SHapley Additive exPlanations (SHAP) were used to interpret the model.

**Results:**

Postoperative FI occurred in 36.04% of patients. LASSO identified nine predictors. The random forest model demonstrated the best balance between discrimination and stability, achieving an AUC of 0.889 (95% CI: 0.847–0.925) in the validation cohort, with good calibration and consistent clinical net benefit. SHAP analysis identified elevated fasting glucose, advanced tumor stage, low serum potassium, longer operative time, and a lower Advanced Lung Cancer Inflammation Index (ALI) as the most influential factors, with nonlinear threshold effects observed for key metabolic variables.

**Conclusion:**

This interpretable model enables accurate perioperative risk stratification for FI using routinely available clinical data. The findings highlight the role of metabolic–inflammatory imbalance and support risk-adapted, individualized nutritional management in patients undergoing oral cancer reconstruction. External validation is warranted.

## 1 Introduction

Oral cancer is one of the most prevalent malignancies of the head and neck (1). For patients with advanced disease, radical resection combined with free flap reconstruction remains the standard approach to restore function and facial integrity (2, 3). These procedures, however, are highly extensive and physiologically demanding. Prolonged operative time and substantial surgical trauma often trigger pronounced metabolic stress and may delay postoperative recovery, placing considerable pressure on perioperative management (4, 5).

Nutritional vulnerability is a defining feature of this population (6). Even before surgery, many patients experience inadequate oral intake due to tumor-related pain, chewing difficulty, and dysphagia (7). Chronic tumor-associated inflammation further promotes catabolism and impairs protein synthesis, thereby accelerating nutritional decline. Previous reports indicate that 30%−50% of patients with head and neck cancer present with preoperative malnutrition (8, 9). After free flap reconstruction, restricted oral intake, swallowing dysfunction, and airway management often necessitate enteral nutrition (EN) *via* a nasogastric tube as the primary nutritional strategy (10, 11).

Although EN preserves gut integrity and supports immune function, feeding intolerance (FI) frequently complicates its implementation (12). FI may manifest as abdominal distension, vomiting, diarrhea, high gastric residual volumes, or failure to achieve target caloric intake (13). These events compromise energy delivery and may prolong hospitalization and increase complication rates (14, 15). In patients undergoing major oral oncologic surgery, who already have limited metabolic reserve and a heightened inflammatory burden, FI may be particularly prevalent and mechanistically complex.

Most existing FI research has focused on critically ill populations in the intensive care unit (ICU), where risk factors are closely linked to organ failure and hemodynamic instability (16–18). Data specific to major head and neck oncologic surgery remain scarce. Patients undergoing free flap reconstruction represent a distinct surgical cohort, as they enter surgery with preexisting malnutrition and chronic inflammation and are subsequently exposed to extensive resection and reconstructive trauma (19–21). The determinants of FI in this setting may therefore differ from those identified in general ICU models, limiting the applicability of previously developed prediction tools.

FI is a multifactorial event shaped by metabolic status, inflammatory activity, electrolyte balance, tumor burden, and perioperative management. Interactions among these variables are often nonlinear. Conventional logistic regression may not fully capture such complexity. Machine learning approaches offer advantages in modeling high-dimensional data and nonlinear relationships (22, 23). When combined with interpretability techniques such as SHapley Additive exPlanations (SHAP), they can provide both predictive accuracy and clinically meaningful interpretation (24, 25).

In this context, we aimed to develop and internally validate an interpretable machine learning model to predict the risk of FI following free flap reconstruction for oral cancer. Using routinely available perioperative clinical indicators, we sought to enable early risk stratification and support proactive, individualized nutritional management.

## 2 Methods

### 2.1 Data sources and study population

This retrospective single-center cohort study included patients who underwent radical resection for oral cancer with simultaneous free flap reconstruction at the Department of Oral and Maxillofacial Surgery, Suining Central Hospital, between August 2017 and August 2025.

Eligible patients were identified through the institutional electronic medical record system. The inclusion criteria were: (1) pathologically confirmed malignant oral tumor; (2) radical resection with concurrent free flap reconstruction; and (3) availability of complete perioperative clinical and laboratory data. Patients were excluded if they had: (1) missing key variables required for model development; (2) perioperative mortality; or (3) pre-existing severe gastrointestinal dysfunction that could interfere with the assessment of EN tolerance.

**Figure 1:**
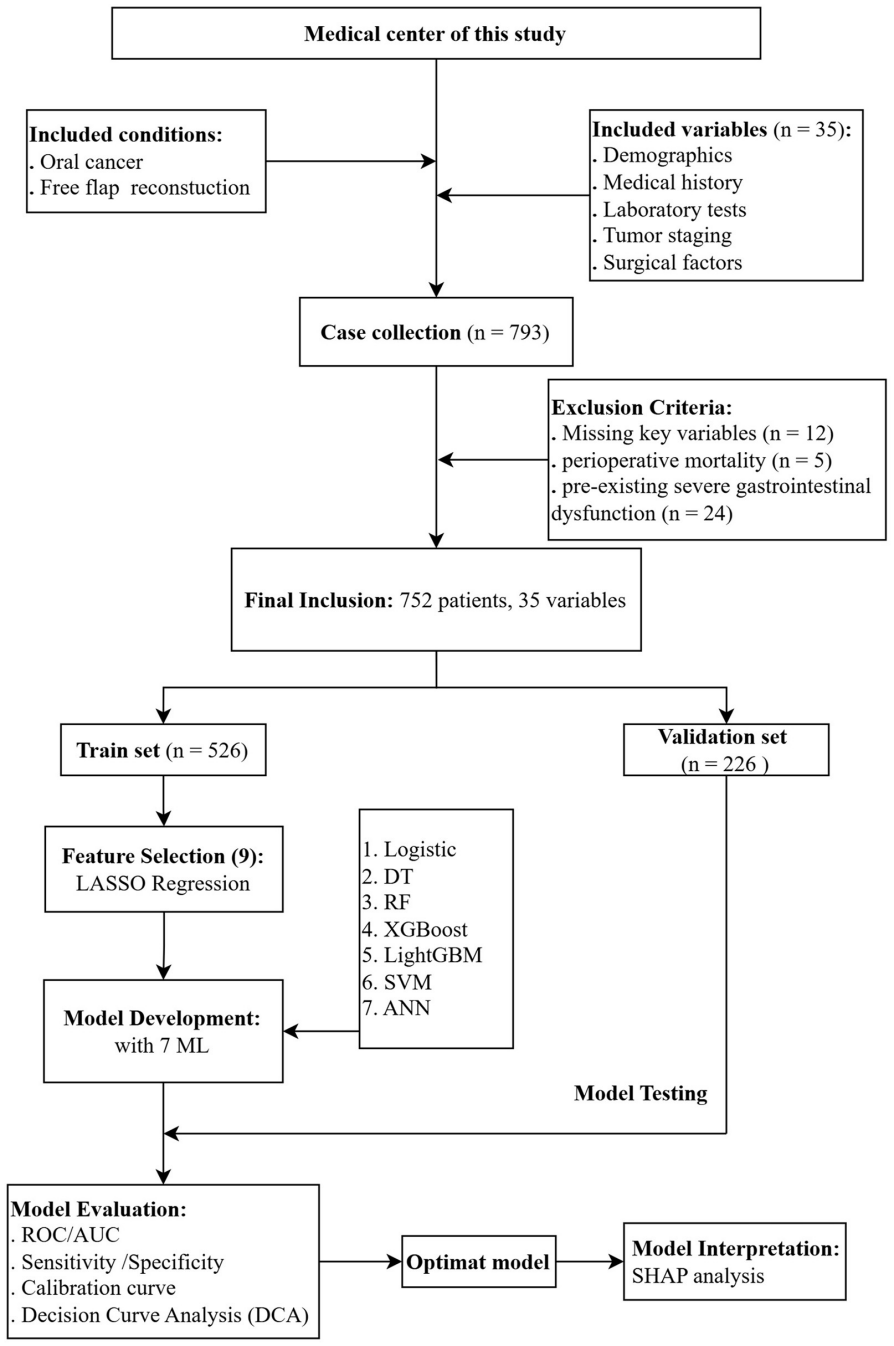
Flowchart of the study design and analytical workflow.

A total of 752 patients met the predefined criteria and were included in the final analysis. The patient selection process is illustrated in Figure 1.

The study protocol was approved by the Biomedical Research Ethics Committee of Suining Central Hospital (Approval No. KYLLKYS20250144). Given the retrospective nature of the study, the requirement for informed consent was waived. All procedures were conducted in accordance with the Declaration of Helsinki, and all patient data were anonymized prior to analysis.

### 2.2 Data collection and variable definition

Clinical data were retrospectively extracted from the institutional electronic medical record system using a predefined standardized case report form. The collected variables included demographic characteristics (age, sex), medical history (smoking, alcohol consumption, hypertension, hyperlipidemia, chronic obstructive pulmonary disease, and cardiovascular disease), tumor characteristics (T stage), surgical factors (operative time, intraoperative blood loss, extensive mandibulectomy, bilateral neck dissection, and delayed extubation), and perioperative laboratory parameters [red blood cell count, hemoglobin, red cell distribution width (RDW), platelet count, mean platelet volume (MPV), alanine aminotransferase (ALT), aspartate aminotransferase (AST), total bilirubin, gamma-glutamyl transferase (GGT), creatinine, uric acid, blood urea nitrogen, fasting blood glucose, sodium, potassium, phosphorus, total calcium, triglycerides, high-density lipoprotein cholesterol (HDL-C), and low-density lipoprotein cholesterol (LDL-C)].

All laboratory measurements were obtained from the most recent preoperative blood tests. The Advanced Lung Cancer Inflammation Index (ALI) was calculated as follows:


$$\begin{array}{l} \text{ALI}=\text{BMI }\left(\text{kg}/{\text{m}}^{2}\right)\times \text{serum albumin }\left(\text{g}/\text{dL}\right)/\text{neutrophil}-\text{to}\\ \;-\text{lymphocyte ratio }\left(\text{NLR}\right), \end{array}$$

where NLR was defined as the the ratio of the absolute neutrophil count to the absolute lymphocyte count (26, 27).

Prior to model development, a predefined criterion was established whereby categorical variables with extremely low prevalence (< 2% of the cohort) would be considered for exclusion to avoid sparse-data bias. However, no variables met this criterion in the present dataset; therefore, all 35 candidate variables were retained and entered into the LASSO feature selection procedure.

We have clarified the data quality control procedure. All variables were independently extracted by two trained investigators and cross-verified. Any discrepancies were resolved through discussion and consensus with a senior investigator to ensure data accuracy and consistency.

A complete-case analysis approach was used for model development. Patients with missing key variables required for outcome definition or model construction were excluded prior to analysis. Given the low proportion and non-random nature of missing data, multiple imputation was not performed.

Delayed extubation (DE) was defined as the absence of immediate endotracheal tube removal at the end of surgery, with transfer to the ICU for continued mechanical ventilation (28). DE was recorded at the completion of surgery as an intraoperative/immediate postoperative variable. FI was assessed after initiation of EN during the early postoperative period, typically within 1–3 days following surgery. Therefore, DE preceded the occurrence of FI in the perioperative timeline and was considered a perioperative predictive variable.

The primary outcome was FI. FI was defined according to the criteria proposed by the European Society of Intensive Care Medicine and included any of the following after initiation of EN: (1) significant gastrointestinal adverse events (e.g., regurgitation or vomiting, aspiration, abdominal distension, diarrhea, constipation, or 24-h gastric residual volume ≥500 mL); (2) failure to achieve an energy target of 20 kcal/kg/day within 72 h; or (3) discontinuation of EN for clinical reasons. The occurrence of any of these criteria was considered indicative of FI (29). The composite endpoint assigned equal weight to each component, therefore FI was defined as the presence of any single criterion, although the components reflect different clinical severities.

### 2.3 Statistical analysis

#### 2.3.1 Comparison of baseline characteristics

A total of 752 patients were included in the final analysis. According to the occurrence of postoperative FI, patients were classified into the FI group and the non-FI group. Baseline characteristics were compared between the two groups.

Categorical variables are presented as frequencies (percentages) and were compared using the chi-square test or Fisher's exact test, as appropriate. Continuous variables were assessed for normality using the Shapiro–Wilk test. As most variables were not normally distributed, they are presented as median (interquartile range [IQR]) and were compared using the Mann–Whitney U test.

All statistical tests were two-tailed, and a *P* value < 0.05 was considered statistically significant.

#### 2.3.2 Model development and validation

The dataset was randomly split into a training set (70%, *n* = 526) and a validation set (30%, *n* = 226) for model development and internal validation, using stratified random sampling based on the outcome variable. This procedure was implemented using the createDataPartition function from the caret package.

To avoid scale-related bias, all continuous variables were standardized using Z-score normalization. Standardization parameters were derived exclusively from the training set and subsequently applied to the validation set to prevent data leakage. Categorical variables were encoded using one-hot encoding.

Feature selection was performed within the training set using Least Absolute Shrinkage and Selection Operator (LASSO) regression with 10-fold cross-validation. The penalty parameter was selected according to the one-standard-error rule (λ0.1se) to enhance model parsimony and generalizability. Variables with non-zero coefficients were retained for model construction.

Based on the selected features, seven machine learning algorithms were developed in the training set: Logistic Regression, Decision Tree, Random Forest, eXtreme Gradient Boosting (XGBoost), Light Gradient Boosting Machine (LightGBM), Support Vector Machine (SVM), and Artificial Neural Network (ANN).

Hyperparameter tuning was performed using grid search with predefined algorithm-specific parameter ranges (Supplementary Table S1) within the training set. Model performance was evaluated using 5-fold cross-validation, and the optimal hyperparameter combination was selected based on the highest mean area under the receiver operating characteristic curve (AUC). To reduce overfitting, model complexity was controlled through parameter constraints and regularization techniques.

Model performance was evaluated in both the training and independent validation sets. Discrimination was assessed using the area under the receiver operating characteristic curve (AUC).Calibration was evaluated using calibration curves and Brier scores. Clinical utility was assessed through decision curve analysis (DCA). Additional performance metrics included accuracy, sensitivity, specificity, F1 score, Cohen's kappa, and Youden index.

To assess potential overfitting, model performance was compared between the training and validation sets, as well as across cross-validation results.

The final model was selected based on a comprehensive assessment of discrimination, calibration, and stability, prioritizing models with robust generalization performance and lower overfitting risk.

#### 2.3.3 Model interpretability

To improve model transparency and quantify the contribution of individual predictors, SHAP was applied for *post-hoc* interpretability analysis. Based on cooperative game theory, SHAP decomposes model predictions into additive feature contributions, enabling quantification of each variable's impact on the predicted risk.

Both global and local interpretability were assessed. At the global level, feature importance was ranked according to the mean absolute SHAP value of each predictor, and a summary plot was generated to visualize the distribution and direction of feature effects. For continuous variables, dependence plots were constructed to illustrate nonlinear associations between predictors and predicted risk.

At the individual level, SHAP values were used to decompose single-patient predictions. Waterfall plots were generated to display the contribution of each variable to the final predicted probability, facilitating interpretation of individual risk profiles.

All statistical analyses and model development were performed using R software (version 4.4.3). SHAP computation and visualization were conducted in Python (version 3.12). All statistical tests were two-sided, with *P* < 0.05 considered statistically significant. The overall study workflow is presented in Figure 1.

#### 2.3.4 Sensitivity analysis

To test the robustness of the primary model, a sensitivity analysis was conducted using a stricter definition of FI, limited to gastrointestinal adverse events after initiation of EN. The same preprocessing steps, including stratified splitting, feature selection, and model training, were applied to this redefined outcome. The best-performing algorithm from the primary analysis was then retrained, and its performance was evaluated and compared with the original model in the validation cohort.

## 3 Results

### 3.1 Baseline characteristics

A total of 752 patients who underwent radical resection for oral cancer with free flap reconstruction were included. Postoperative FI occurred in 271 patients (36.04%), while 481 patients (63.96%) tolerated EN. Within the FI cohort, gastrointestinal adverse events accounted for 78.97% (214/271), caloric failure for 20.66% (56/271), and EN interruption for 16.61% (45/271). Baseline characteristics are presented in Table 1.

**Table 1:** Baseline characteristics of patients according to the occurrence of postoperative feeding intolerance (FI).

Variables	Total (*n* = 752)	Tolerance group (*n* = 481)	Intolerance group (*n* = 271)	*P*
Age, M (Q1, Q3)	59.50 (51.00, 67.00)	56.00 (48.00, 66.00)	63.00 (55.00, 68.00)	< 0.001
Surgery time, M (Q1, Q3)	412.00 (380.00, 451.25)	401.00 (380.00, 431.00)	432.00 (381.00, 482.00)	< 0.001
Blood loss, M (Q1, Q3)	375.00 (294.00, 420.00)	356.00 (283.00, 408.00)	387.00 (310.00, 457.50)	< 0.001
RBC, M (Q1, Q3)	4.66 (4.34, 4.97)	4.63 (4.32, 4.98)	4.68 (4.39, 4.96)	0.287
Hemoglobin, M (Q1, Q3)	14.10 (13.10, 15.10)	14.10 (13.10, 15.10)	14.10 (13.30, 15.10)	0.591
RDW, M (Q1, Q3)	13.50 (13.10, 14.10)	13.40 (13.00, 14.00)	13.60 (13.10, 14.20)	0.072
Platelet, M (Q1, Q3)	225.00 (189.00, 270.25)	227.00 (192.00, 267.00)	222.00 (183.50, 273.00)	0.348
MPV, M (Q1, Q3)	8.40 (7.80, 9.00)	8.30 (7.70, 8.90)	8.50 (7.90, 9.10)	0.013
ALT, M (Q1, Q3)	21.00 (16.00, 28.00)	21.00 (17.00, 28.00)	21.00 (16.00, 28.00)	0.735
AST, M (Q1, Q3)	23.00 (19.00, 27.00)	23.00 (19.00, 27.00)	23.00 (19.00, 27.00)	0.398
Total bilirubin, M (Q1, Q3)	10.26 (8.55, 13.68)	10.26 (8.55, 13.68)	10.26 (8.55, 13.68)	0.903
GGT, M (Q1, Q3)	20.00 (15.00, 31.00)	20.00 (15.00, 32.00)	20.00 (15.00, 31.00)	0.774
Creatinine, M (Q1, Q3)	74.26 (62.76, 89.28)	74.26 (61.88, 89.28)	73.37 (62.76, 90.17)	0.689
Uric acid, M (Q1, Q3)	321.20 (267.70, 380.70)	315.20 (267.70, 374.70)	327.10 (267.70, 392.60)	0.121
Blood urea nitrogen, M (Q1, Q3)	4.64 (3.57, 5.71)	4.64 (3.57, 5.71)	4.64 (3.57, 6.07)	0.248
Fasting blood glucose, M (Q1, Q3)	5.25 (4.92, 5.89)	5.14 (4.86, 5.53)	5.64 (5.05, 11.11)	< 0.001
Sodium, M (Q1, Q3)	140.00 (139.00, 141.00)	140.00 (139.00, 141.00)	140.00 (139.00, 141.00)	0.915
Potassium, M (Q1, Q3)	4.11 (3.94, 4.29)	4.14 (3.98, 4.29)	4.06 (3.81, 4.28)	< 0.001
Phosphorus, M (Q1, Q3)	1.20 (1.10, 1.32)	1.20 (1.10, 1.32)	1.16 (1.07, 1.31)	0.083
Total calcium, M (Q1, Q3)	2.35 (2.30, 2.40)	2.35 (2.30, 2.40)	2.35 (2.30, 2.40)	0.816
Triglycerides, M (Q1, Q3)	1.16 (0.79, 1.73)	1.16 (0.81, 1.71)	1.17 (0.78, 1.80)	0.734
HDL-C, M (Q1, Q3)	1.37 (1.14, 1.66)	1.37 (1.16, 1.68)	1.32 (1.09, 1.63)	0.078
LDL-C, M (Q1, Q3)	2.95 (2.35, 3.54)	2.97 (2.40, 3.57)	2.85 (2.31, 3.46)	0.113
ALI, M (Q1, Q3)	57.80 (40.65, 71.97)	60.00 (45.26, 73.00)	51.69 (34.41, 69.52)	< 0.001
Delayed extubation, *n* (%)				< 0.001
No	510 (67.82)	376 (78.17)	134 (49.45)	
Yes	242 (32.18)	105 (21.83)	137 (50.55)	
Sex, *n* (%)				0.123
Male	430 (57.18)	265 (55.09)	165 (60.89)	
Female	322 (42.82)	216 (44.91)	106 (39.11)	
Smoking status, *n* (%)				< 0.001
No	411 (54.65)	289 (60.08)	122 (45.02)	
Yes	341 (45.35)	192 (39.92)	149 (54.98)	
Alcohol intake, *n* (%)				0.930
No	537 (71.41)	344 (71.52)	193 (71.22)	
Yes	215 (28.59)	137 (28.48)	78 (28.78)	
Hypertension, *n* (%)				0.810
No	373 (49.60)	237 (49.27)	136 (50.18)	
Yes	379 (50.40)	244 (50.73)	135 (49.82)	
Hyperlipidemia, *n* (%)				0.769
No	185 (24.60)	120 (24.95)	65 (23.99)	
Yes	567 (75.40)	361 (75.05)	206 (76.01)	
COPD, *n* (%)				0.654
No	721 (95.88)	460 (95.63)	261 (96.31)	
Yes	31 (4.12)	21 (4.37)	10 (3.69)	
CVD, *n* (%)				0.705
No	673 (89.49)	432 (89.81)	241 (88.93)	
Yes	79 (10.51)	49 (10.19)	30 (11.07)	
Tumor T stage, *n* (%)				< 0.001
T1/T2	414 (55.05)	322 (66.94)	92 (33.95)	
T3/T4	338 (44.95)	159 (33.06)	179 (66.05)	
Extensive maxillomandibular resection, *n* (%)				< 0.001
No	532 (70.74)	379 (78.79)	153 (56.46)	
Yes	220 (29.26)	102 (21.21)	118 (43.54)	
Bilateral neck dissection, *n* (%)				0.014
No	597 (79.39)	395 (82.12)	202 (74.54)	
Yes	155 (20.61)	86 (17.88)	69 (25.46)	
GI adverse events, *n* (%)				< 0.001
No	538 (71.54)	481 (100)	57 (21.03)	
Yes	214 (28.46)	0 (0.00)	214 (78.97)	
Caloric failure, *n* (%)				< 0.001
No	696 (92.55)	481 (100)	215 (79.34)	
Yes	56 (7.45)	0 (0.00)	56 (20.66)	
EN interruption, *n* (%)				< 0.001
No	707 (94.02)	481(100)	226 (83.39)	
Yes	45 (5.98)	0 (0.00)	45 (16.61)	

Data are presented as median [interquartile range (Q1, Q3)] or number (percentage). Units: age (years); operative time (min); intraoperative blood loss (mL); RBC ( × 10^12^/L); hemoglobin (g/dL); RDW (%); platelet ( × 10^9^/L); MPV (fL); ALT (U/L); AST (U/L); total bilirubin (μmol/L); GGT (U/L); creatinine (μmol/L); uric acid (μmol/L); blood urea nitrogen (mmol/L); fasting blood glucose (mmol/L); sodium, potassium, phosphorus (mmol/L); total calcium (mmol/L); triglycerides, HDL-C, LDL-C (mmol/L); ALI (advanced lung cancer inflammation index). P values were calculated using the Mann–Whitney U test for continuous variables and the chi-square test or Fisher's exact test, as appropriate.

Patients who developed FI were older and had longer operative duration and greater intraoperative blood loss compared with those who tolerated EN. Advanced tumor stage (T3/T4), extensive mandibulectomy, and bilateral neck dissection were also more common in the FI group, reflecting a higher surgical complexity and disease burden. In addition, smoking history and delayed extubation were more frequently observed among patients with FI, suggesting a more adverse perioperative condition in this population.

Regarding laboratory parameters, patients with FI showed higher preoperative fasting blood glucose levels and lower serum potassium levels, along with slightly increased mean platelet volume. The Advanced Lung Cancer Inflammation Index (ALI) was lower in the FI group, indicating a combination of reduced nutritional reserve and higher inflammatory burden. No obvious differences were observed in most hematological, hepatic, renal, or comorbidity-related variables between groups.

Overall, these baseline differences suggest that patients who developed FI tended to present with more advanced disease stage, greater surgical trauma, and less favorable metabolic and nutritional status. These findings primarily reflect baseline perioperative clinical status rather than isolated statistical differences.

### 3.2 Development and performance of machine learning models

#### 3.2.1 Data partition and balance assessment

All patients were randomly assigned to a training set (*n* = 526) and a validation set (*n* = 226). The training set was used for model development, and the validation set for independent performance evaluation. Baseline characteristics of both cohorts are presented in Supplementary Table S2.

The distribution of demographic variables, perioperative factors, and laboratory parameters was comparable between the two cohorts. The incidence of postoperative FI did not differ significantly between the training and validation sets (35.55 vs. 37.17%, *P* = 0.672). Key variables, including age, operative time, intraoperative blood loss, tumor T stage, extensive mandibulectomy, bilateral neck dissection, delayed extubation, fasting blood glucose, serum potassium, and ALI, showed no statistically significant differences between groups (all *P* > 0.05).

Although statistically significant differences were observed in low-density lipoprotein cholesterol, serum sodium, and the prevalence of chronic obstructive pulmonary disease, the absolute differences were small.

#### 3.2.2 Feature selection

**Figure 2:**
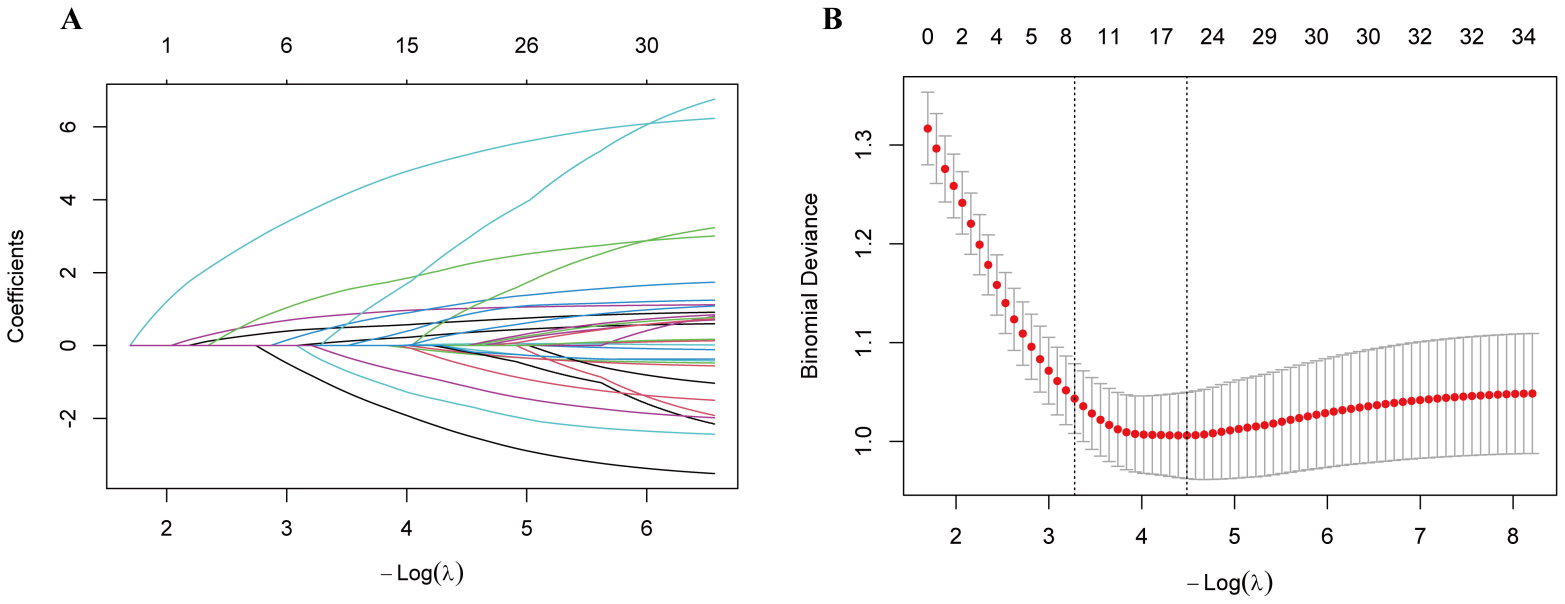
Multidimensional analyses of variables associated with feeding intolerance (FI). **(A)** Least Absolute Shrinkage and Selection Operator (LASSO) coefficient profiles plotted against the penalty parameter (λ), illustrating the shrinkage process of candidate predictors. **(B)** Ten-fold cross-validation for optimal λ selection (λ0.1se), identifying the most relevant predictors associated with FI.

Feature selection was conducted using LASSO regression with 10-fold cross-validation in the training set. The optimal penalty parameter was selected according to the one-standard-error criterion (λ0.1se = 0.0377), corresponding to the minimum binomial deviance within one standard error (Figure 2).

At this λ value, nine variables retained non-zero coefficients and were selected for model development. The selected predictors were, in descending order of absolute coefficient magnitude: fasting blood glucose, age, serum potassium, tumor T stage, operative time, delayed extubation, low-density lipoprotein cholesterol, ALI, and extensive mandibulectomy.

#### 3.2.3 Model evaluation

**Figure 3:**
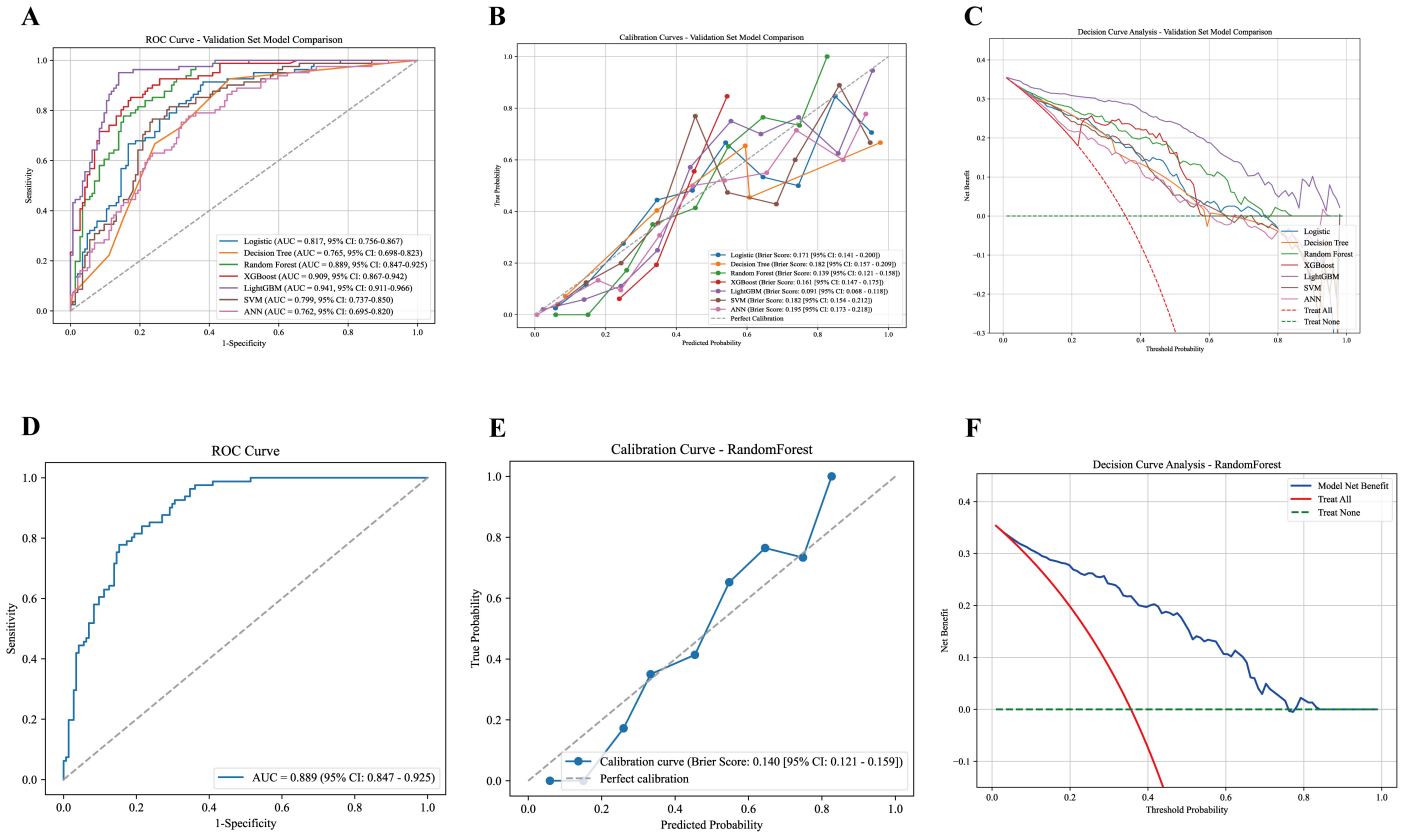
Performance evaluation of machine learning models in the validation set. **(A–C)** Receiver operating characteristic (ROC) curves, calibration curves, and decision curve analysis (DCA) of seven candidate models. **(D–F)** ROC curve, calibration curve (Brier score = 0.140), and decision curve analysis of the selected random forest (RF) model, demonstrating its predictive performance and net clinical benefit across a range of threshold probabilities.

Model performance was evaluated on the independent validation set (Table 2). ROC curves, calibration curves, and decision curve analyses are presented in Figures 3A–C.

**Table 2:** Predictive performance of seven machine learning models in the training and validation sets.

Model	AUC (95% CI)	Accuracy	Precision	Sensitivity	Specificity	F1 score	Kappa	Youden's J	NPV
validation sets									
Logistic	0.817 (0.756–0.867)	0.742	0.667	0.568	0.840	0.613	0.422	0.408	0.776
Decision tree	0.765 (0.698–0.823)	0.724	0.607	0.667	0.757	0.635	0.415	0.424	0.801
Random Forest	0.889 (0.847–0.925)	0.804	0.740	0.704	0.861	0.722	0.571	0.565	0.838
XGBoost	0.909 (0.867–0.942)	0.800	0.846	0.543	0.944	0.662	0.529	0.488	0.786
LightGBM	0.941 (0.911–0.966)	0.880	0.807	0.877	0.882	0.840	0.744	0.758	0.927
SVM	0.799 (0.737–0.850)	0.698	0.600	0.481	0.819	0.534	0.315	0.301	0.738
ANN	0.762 (0.695–0.820)	0.698	0.592	0.519	0.799	0.553	0.326	0.317	0.747
Training sets									
Logistic	0.841 (0.806–0.874)	0.780	0.750	0.584	0.890	0.657	0.498	0.474	0.792
Decision tree	0.804 (0.763–0.840)	0.751	0.656	0.653	0.807	0.654	0.460	0.460	0.805
Random forest	0.963 (0.949–0.975)	0.886	0.874	0.800	0.935	0.835	0.748	0.735	0.892
XGBoost	0.987 (0.978–0.994)	0.867	1.000	0.632	1.000	0.774	0.687	0.632	0.828
LightGBM	1.000 (1.000–1.000)	0.998	1.000	0.995	1.000	0.997	0.996	0.995	0.997
SVM	0.843 (0.807–0.877)	0.776	0.786	0.521	0.920	0.627	0.476	0.441	0.773
ANN	0.810 (0.771–0.849)	0.757	0.718	0.537	0.881	0.614	0.443	0.418	0.771

AUC, area under the receiver operating characteristic curve; CI, confidence interval; NPV, negative predictive value; SVM, support vector machine; ANN, artificial neural network; XGBoost, extreme gradient boosting; LightGBM, light gradient boosting machine.

All candidate models achieved AUC values above 0.75 in the validation cohort. The LightGBM model showed the highest AUC (0.941, 95% CI: 0.911–0.966), with an accuracy of 0.880 and an F1 score of 0.840. The XGBoost model achieved an AUC of 0.909 (95% CI: 0.867–0.942), with an accuracy of 0.800 and an F1 score of 0.662. The Random Forest model yielded an AUC of 0.889 (95% CI: 0.847–0.925), with an accuracy of 0.804, sensitivity of 0.704, specificity of 0.861, and an F1 score of 0.722. Logistic regression achieved an AUC of 0.817 (95% CI: 0.756–0.867), while decision tree, support vector machine, and artificial neural network models demonstrated AUC values of 0.765, 0.799, and 0.762, respectively.

Calibration curves indicated acceptable agreement between predicted probabilities and observed outcomes, particularly for ensemble-based models (Figure 3B). Among these, the Random Forest model showed a lower Brier score (0.139) compared with XGBoost (0.161), suggesting improved calibration. Decision curve analysis demonstrated positive net benefit across a threshold probability range of approximately 0.05–0.75 for most models, exceeding both the “treat-all” and “treat-none” strategies (Figure 3C). The Random Forest model showed a wider net benefit range compared with XGBoost (approximately 0.05–0.85 vs. 0.21–0.59), indicating broader clinical utility.

Model comparison was performed using a comprehensive framework that considered discrimination, calibration, and clinical utility, rather than AUC alone.

Although the LightGBM model achieved the highest discrimination in the validation cohort, its perfect training performance (AUC = 1.000) suggested higher model complexity and potential overfitting.

In contrast, the Random Forest model demonstrated more balanced performance between training and validation cohorts, together with stable calibration and clinical utility, and was therefore selected for further interpretation (Figures 3D–F), showing an AUC of 0.889 (95% CI: 0.847–0.925).

#### 3.2.4 Model interpretation

**Figure 4:**
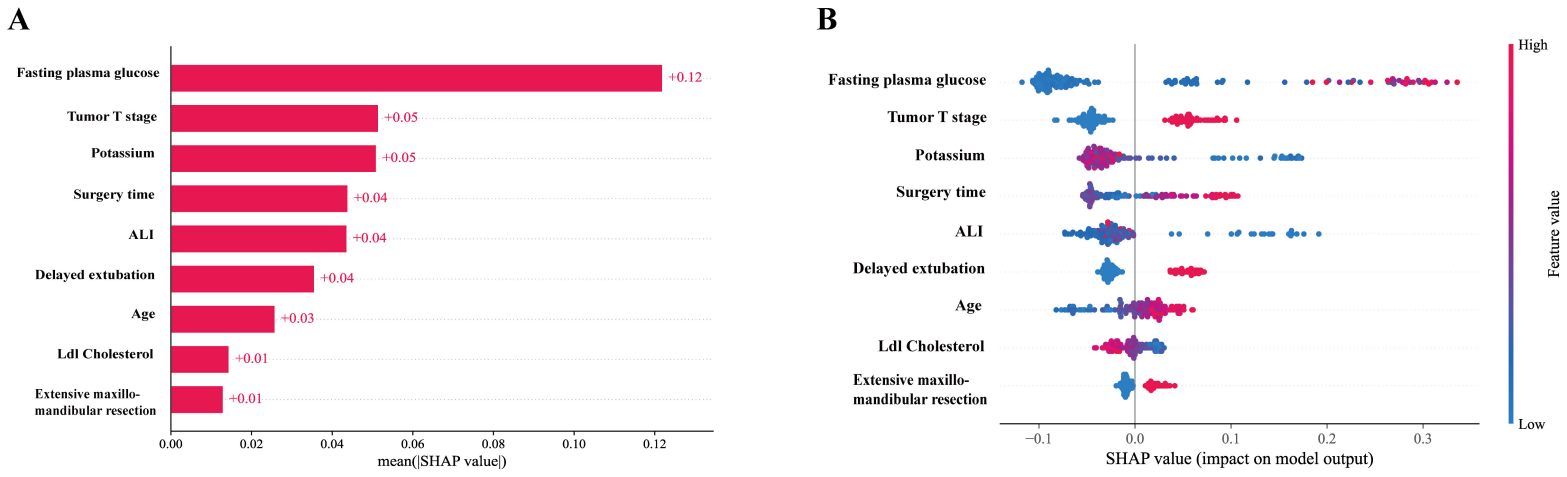
SHAP-based feature importance analysis of the RF model. **(A)** Feature importance ranking based on mean absolute SHAP values, representing each predictor's overall contribution to model predictions. **(B)** SHAP summary plot showing both the magnitude and direction of each feature's effect on the predicted risk of FI.

SHAP were applied to interpret the final random forest model. Global feature importance is presented in Figure 4A. Fasting blood glucose showed the highest mean absolute SHAP value (0.12), contributing most to model predictions. Tumor T stage (0.05), serum potassium (0.05), operative time (0.04), ALI (0.04), and delayed extubation (0.04) followed. Age (0.03), LDL cholesterol (0.01), and extensive mandibulectomy (0.01) had relatively smaller contributions.

The SHAP summary plot (Figure 4B) illustrates both the direction and magnitude of feature effects. Higher fasting blood glucose levels were associated with positive SHAP values. Lower serum potassium and lower ALI were similarly linked to increased predicted risk. Advanced tumor stage and extensive mandibulectomy showed predominantly positive SHAP distributions. Increasing age and longer operative time were also associated with higher SHAP values.

**Figure 5:**
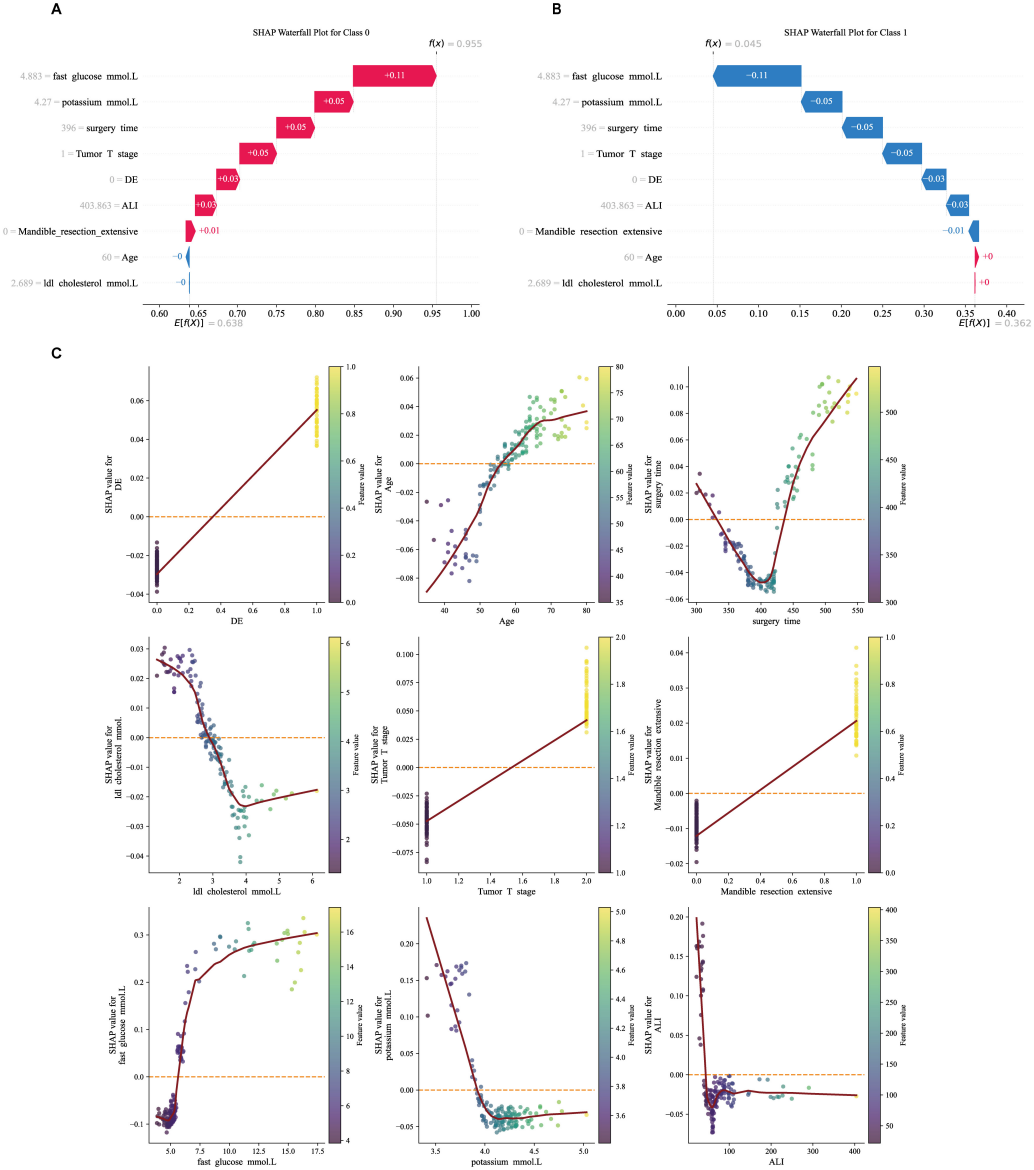
SHAP-based interpretability of the RF model. **(A, B)** Waterfall plots showing individual-level prediction decomposition for representative cases of feeding intolerance and tolerance. **(C)** SHAP dependence plots illustrating nonlinear associations between key predictors and model output.

Waterfall plots (Figures 5A, B) demonstrate individual-level prediction decomposition. In patients predicted to tolerate EN, lower fasting blood glucose contributed most strongly toward reduced risk, followed by higher ALI and shorter operative time. In contrast, higher tumor stage and delayed extubation shifted predictions toward intolerance.

Dependency plots (Figure 5C) further characterize nonlinear relationships between continuous predictors and model output. Fasting blood glucose displayed a nonlinear upward trend, with SHAP values increasing markedly beyond approximately 5.5 mmol/L. Serum potassium showed an inverse nonlinear pattern, with higher SHAP values observed below approximately 4.0 mmol/L. ALI demonstrated a monotonic decreasing relationship, with diminishing SHAP values at higher levels. Operative time and age exhibited gradual risk accumulation patterns as values increased. Binary variables coded as “yes” generally corresponded to positive SHAP contributions.

### 3.3 Sensitivity analysis

A sensitivity analysis was performed by redefining FI as gastrointestinal adverse events only. The same modeling strategy was applied to retrain the random forest model. In the training cohort, the random forest model achieved an AUC of 0.859. The Brier score was 0.147. Decision curve analysis indicated net clinical benefit across threshold probabilities ranging from 0.1 to 0.7. Performance metrics for all models are presented in Supplementary Table S3. The ROC curve, calibration curve, and decision curve analysis of the random forest model are shown in Supplementary Figure S1.

## 4 Discussion

In this single-center cohort of 752 patients undergoing free flap reconstruction for oral cancer, we developed and internally validated an interpretable machine learning model for preoperative prediction of postoperative enteral FI. The overall incidence of FI was 36.04%, underscoring its clinical relevance in this high-risk surgical population. Evidence regarding postoperative FI in this population remains limited, with only one previous study reporting an incidence of approximately 42.6% (30). Our observed incidence of 36.04% is broadly comparable with this report, and the slight difference may be related to variations in sample size, patient characteristics, and perioperative management strategies. Among the candidate algorithms, the random forest model achieved robust discrimination (AUC = 0.889) and good calibration in the independent validation set, with consistent net benefit across clinically relevant threshold probabilities. SHAP analysis identified fasting blood glucose, tumor T stage, serum potassium, operative time, and ALI as the principal contributors to model output. Nonlinear patterns were observed for several continuous variables, particularly fasting blood glucose and serum potassium, suggesting threshold effects rather than simple linear associations. Collectively, these findings suggest that FI in this setting may be associated with perioperative disturbances in metabolic and inflammatory homeostasis, which manifest clinically as impaired gastrointestinal tolerance. Rather than representing an isolated motility disorder, FI may reflect a broader systemic response to tumor burden and surgical stress.

These findings should be interpreted in the context of the perioperative physiological characteristics specific to patients with oral cancer. Compared with general critically ill populations, these patients frequently exhibit compromised nutritional reserves before surgery due to tumor-related oral intake limitation and chronic systemic inflammation (31). Radical tumor resection combined with free flap reconstruction further imposes substantial acute surgical stress (32, 33). The combination of limited preoperative reserve and pronounced intraoperative trauma may increase vulnerability of gastrointestinal function during the early postoperative period (34). In this setting, FIlikely reflects the interaction between baseline metabolic fragility and surgical stress response (35). Therefore, the development of population-specific risk prediction tools may facilitate early identification of high-risk individuals and support more individualized perioperative nutritional management strategies.

From a mechanistic standpoint, the present findings suggest that preoperative metabolic and nutritional status may reflect baseline physiological vulnerability associated with the development of postoperative FI in oral cancer patients undergoing free flap reconstruction (20, 36). Elevated preoperative fasting blood glucose was associated with higher model-predicted risk, with a nonlinear pattern observed around approximately 5.5 mmol/L. This may reflect underlying metabolic stress and insulin resistance that have been reported in surgical and oncological populations, and which are potentially associated with impaired gastrointestinal function (37, 38). In patients with oral cancer requiring free flap reconstruction, this may reflect chronic tumor-related metabolic stress and reduced physiological reserve, rather than an acute perioperative response. Reduced serum potassium levels, particularly below 4.0 mmol/L, highlight the importance of electrolyte balance in maintaining gastrointestinal smooth muscle excitability and coordinated peristalsis (39). In this surgical population, such abnormalities are often observed preoperatively and may be associated with reduced oral intake, swallowing dysfunction, and peri-tumor nutritional impairment. In parallel, lower ALI values indicate a combined state of systemic inflammation and limited nutritional reserve, which may weaken intestinal barrier integrity and recovery capacity in the postoperative setting (40). In oral cancer patients undergoing extensive resection and free flap reconstruction, this likely reflects chronic tumor-related inflammation combined with preoperative nutritional depletion. Together, these factors may represent interrelated components of apreoperative physiological vulnerability specific to oral cancer patients undergoing major reconstructive surgery rather than isolated metabolic abnormalities. However, given the observational design of this study, causal pathways cannot be established and warrant further prospective investigation.

Higher tumor T stage and longer operative time were also associated with increased FI risk (41). These variables may reflect greater tumor burden and procedural complexity, respectively, and suggest the cumulative physiological stress imposed by advanced disease and extensive surgery (42). When integrated with metabolic indicators, they allow the model to capture a broader risk continuum spanning baseline vulnerability and treatment-related stress exposure. The observed association with delayed extubation further suggests that postoperative respiratory management and sedation depth may be linked to gastrointestinal recovery (43). However, the underlying mechanisms remain to be clarified.

The clinical relevance of this study lies in shifting perioperative management of FI from reactive treatment to early risk identification and prevention. Because all predictors are readily available preoperatively or in the immediate postoperative period, risk assessment can be performed before initiation of EN. Patients were classified into high- and low-risk groups according to the optimal probability cutoff derived from the Youden index of the ROC curve in the validation cohort. For patients in the high-risk group, a more proactive and structured perioperative strategy may be warranted. Blood glucose should be kept under closer control, with a practical target around or below 5.5 mmol/L when clinically feasible. Serum potassium should be monitored and corrected promptly to maintain levels above 4.0 mmol/L. EN should be started at a lower rate and advanced more cautiously, with closer observation during the first 48–72 h to assess gastrointestinal tolerance. Lower ALI values may help further refine risk identification, reflecting a combination of inflammatory burden and reduced nutritional reserve. While ALI itself is not a direct treatment target, its components may be improved indirectly through nutritional support and timely management of postoperative inflammatory or infectious complications. For low-risk patients, standard postoperative feeding protocols with routine monitoring and gradual advancement of EN appear appropriate. Although these strategies need confirmation in prospective studies, they provide a workable framework for translating model-based risk stratification into clinical decision-making in patients undergoing oral cancer free flap reconstruction.

From a methodological perspective, this study prioritized generalizability and stability over optimal training performance. Although multiple strategies were applied to mitigate overfitting, including hyperparameter tuning, regularization, and cross-validation, residual overfitting remained in certain high-capacity models. In particular, the LightGBM model achieved a perfect AUC of 1.000 in the training set, suggesting a higher degree of model complexity and potential overfitting tendency despite its strong performance in the validation cohort. In contrast, the random forest model demonstrated more balanced performance between the training and validation sets, indicating more stable learning behavior and reduced sensitivity to data noise. Therefore, considering both predictive performance and model stability, the random forest model was selected as the final model. The use of SHAP further improved model transparency, enabling interpretation of variable contributions and enhancing clinical interpretability.

This study has several limitations. First, as a single-center retrospective analysis, the model's generalizability requires validation in external multicenter cohorts. In addition, although an independent validation cohort together with cross-validation was used for internal evaluation, the findings should still be interpreted as internally validated results, and potential optimism bias cannot be completely excluded. Second, the variables included were largely routine clinical indicators; future studies may incorporate dynamic nutritional data, objective measures of gastrointestinal motility, or additional biomarkers to develop a more comprehensive predictive framework. Third, given the retrospective observational design, the associations identified between predictors and FI should not be interpreted as causal relationships. These associations may also be influenced by residual confounding related to underlying disease severity and perioperative physiological stress. Prospective multicenter validation and interventional studies are warranted to determine whether risk-stratified management strategies can improve nutritional adequacy and related clinical outcomes.

In summary, this study provides evidence that FI after oral cancer free flap reconstruction is closely linked to the interplay between baseline metabolic vulnerability and perioperative inflammatory stress. By applying an explainable machine learning framework, we identified nonlinear risk patterns and key predictors within this complex physiological context. These findings underscore the importance of integrating metabolic, inflammatory, and procedural factors when evaluating postoperative nutritional tolerance and highlight the need for population-specific risk stratification strategies in surgical oncology.

## 5 Conclusion

This study demonstrates that FI is common after oral cancer free flap reconstruction. A machine learning model based on routinely available perioperative variables achieved robust predictive performance and identified key metabolic and inflammatory contributors. These findings support early risk stratification and individualized nutritional management in this high-risk surgical population. Prospective multicenter validation is warranted.

## Data Availability

The original contributions presented in the study are included in the article/Supplementary material, further inquiries can be directed to the corresponding authors.
