# Supplementary material for: Risk stratification of postoperative enteral feeding intolerance using explainable machine learning in oral cancer free flap reconstruction
Source: Front Nutr. 2026 Jun 4;13:1815516. doi: 10.3389/fnut.2026.1815516 (PMC13275280; doi:10.3389/fnut.2026.1815516)
Supplement: Supplementary file 1 [file Table_1.docx]

**Supplementary Material**

**Table S1. Hyperparameter tuning strategy and optimal parameters for machine learning models**

| **Model** | **Hyperparameter search range** | **Optimal parameters** |
| --- | --- | --- |
| Decision Tree | max_depth: [3, 5, 8]; min_samples_split: [8, 12]; max_features: ['sqrt']; ccp_alpha: [0.0, 0.005, 0.01] | {'ccp_alpha': 0.0, 'max_depth': 3, 'max_features': 'sqrt', 'min_samples_split': 8} |
| Random Forest | n_estimators: [100, 200, 300]; max_features: ['sqrt', 2–√p]; max_depth: [5, 8, 10]; min_samples_split: [15, 25]; min_samples_leaf: [5, 10]; max_samples: [0.8, 0.9] | n_estimators = 100 , max_features = 2 |
| XGBoost | learning_rate: [0.01, 0.1, 0.2]; n_estimators: [50, 100, 200]; max_depth: [3, 5, 8]; subsample: [0.8, 0.9, 1.0]; colsample_bytree: [0.8, 1.0]; gamma: [0, 0.1, 0.2]; reg_lambda: [1, 2, 5] | {'colsample_bytree': 1.0, 'gamma': 0.2, 'learning_rate': 0.01, 'max_depth': 8, 'n_estimators': 50, 'reg_lambda': 1, 'subsample': 0.8} |
| LightGBM | learning_rate: [0.1, 0.2]; num_leaves: [15, 31]; n_estimators: [100]; subsample: [0.8, 0.9]; colsample_bytree: [0.8, 0.9]; max_depth: [5, 8]; min_child_samples: [8, 15]; reg_alpha: [0.1, 0.5]; reg_lambda: [1, 2]; min_split_gain: [0.1, 0.2] | {'colsample_bytree': 0.9, 'learning_rate': 0.2, 'max_depth': 5, 'min_child_samples': 8, 'min_split_gain': 0.2, 'n_estimators': 100, 'num_leaves': 15, 'random_state': 123, 'reg_alpha': 0.1, 'reg_lambda': 1, 'subsample': 0.8, 'verbosity': -1} |
| SVM | C: [0.01, 0.1, 1.0]; kernel: ['linear', 'rbf']; gamma: ['scale', 'auto'] | {'C': 0.1, 'gamma': 'scale', 'kernel': 'linear'} |
| ANN | hidden_layer_sizes: [(10,), (25,), (50,), (10,10)]; activation: ['relu', 'tanh']; alpha: [0.001, 0.01, 0.1]; learning_rate_init: [0.001, 0.01]; max_iter: [200] | {'activation': 'relu', 'alpha': 0.01, 'hidden_layer_sizes': (25,), 'learning_rate_init': 0.001, 'max_iter': 200} |

**Table S2. Comparison of Baseline Characteristics Between the Training and validation Sets**

| **Variables** | **Total**  **(n = 752)** | **validation Set**  **(n = 226)** | **Training Set**  **(n = 526)** | ***P*** |
| --- | --- | --- | --- | --- |
|  |  |  |  |  |
| Age, M (Q₁, Q₃) | 59.50 (51.00, 67.00) | 60.00 (51.00, 67.00) | 59.00 (51.00, 66.00) | 0.657 |
| Surgery Time, M (Q₁, Q₃) | 412.00 (380.00, 451.25) | 411.00 (380.00, 449.50) | 414.00 (380.00, 453.50) | 0.599 |
| Blood Loss, M (Q₁, Q₃) | 375.00 (294.00, 420.00) | 380.00 (304.00, 420.00) | 364.00 (290.00, 420.00) | 0.240 |
| RBC, M (Q₁, Q₃) | 4.66 (4.34, 4.97) | 4.62 (4.32, 4.94) | 4.67 (4.37, 4.99) | 0.387 |
| Hemoglobin, M (Q₁, Q₃) | 14.10 (13.10, 15.10) | 14.10 (13.10, 15.07) | 14.20 (13.12, 15.10) | 0.753 |
| RDW, M (Q₁, Q₃) | 13.50 (13.10, 14.10) | 13.40 (13.10, 14.10) | 13.50 (13.00, 14.10) | 0.592 |
| Platelet, M (Q₁, Q₃) | 225.00 (189.00, 270.25) | 217.50 (188.00, 263.75) | 227.00 (189.00, 274.00) | 0.267 |
| MPV, M (Q₁, Q₃) | 8.40 (7.80, 9.00) | 8.40 (8.00, 9.10) | 8.40 (7.70, 8.90) | 0.053 |
| ALT, M (Q₁, Q₃) | 21.00 (16.00, 28.00) | 21.00 (16.00, 28.75) | 21.00 (17.00, 28.00) | 0.988 |
| AST, M (Q₁, Q₃) | 23.00 (19.00, 27.00) | 23.00 (19.00, 28.00) | 23.00 (19.00, 27.00) | 0.843 |
| Bilirubin Total, M (Q₁, Q₃) | 10.26 (8.55, 13.68) | 10.26 (8.55, 13.68) | 10.26 (8.55, 13.68) | 0.548 |
| GGT, M (Q₁, Q₃) | 20.00 (15.00, 31.00) | 20.00 (15.00, 31.00) | 20.00 (15.00, 31.00) | 0.767 |
| Creatinine, M (Q₁, Q₃) | 74.26 (62.76, 89.28) | 74.26 (61.88, 90.17) | 74.26 (62.76, 88.40) | 0.845 |
| Uric Acid, M (Q₁, Q₃) | 321.20 (267.70, 380.70) | 315.20 (261.70, 368.80) | 321.20 (267.70, 386.60) | 0.379 |
| Blood Urea Nitrogen, M (Q₁, Q₃) | 4.64 (3.57, 5.71) | 4.28 (3.21, 5.71) | 4.64 (3.57, 5.71) | 0.078 |
| Fasting plasma glucose, M (Q₁, Q₃) | 5.25 (4.92, 5.89) | 5.29 (4.98, 5.95) | 5.24 (4.90, 5.84) | 0.183 |
| Sodium, M (Q₁, Q₃) | 140.00 (139.00, 141.00) | 140.00 (139.00, 141.00) | 140.00 (139.00, 141.00) | 0.048 |
| Potassium, M (Q₁, Q₃) | 4.11 (3.94, 4.29) | 4.12 (3.94, 4.29) | 4.11 (3.92, 4.29) | 0.903 |
| Phosphorus, M (Q₁, Q₃) | 1.20 (1.10, 1.32) | 1.20 (1.10, 1.32) | 1.20 (1.07, 1.32) | 0.227 |
| Calcium Total, M (Q₁, Q₃) | 2.35 (2.30, 2.40) | 2.35 (2.27, 2.40) | 2.35 (2.30, 2.40) | 0.154 |
| Triglycerides, M (Q₁, Q₃) | 1.16 (0.79, 1.73) | 1.16 (0.82, 1.77) | 1.16 (0.79, 1.68) | 0.412 |
| Hdl Cholesterol, M (Q₁, Q₃) | 1.37 (1.14, 1.66) | 1.32 (1.10, 1.68) | 1.39 (1.16, 1.66) | 0.150 |
| Ldl Cholesterol, M (Q₁, Q₃) | 2.95 (2.35, 3.54) | 2.82 (2.28, 3.34) | 2.97 (2.43, 3.57) | 0.014 |
| ALI, M (Q₁, Q₃) | 57.80 (40.65, 71.97) | 57.89 (41.05, 74.02) | 57.59 (40.17, 71.86) | 0.538 |
| FI, n(%) |  |  |  | 0.672 |
| No | 481 (63.96) | 142 (62.83) | 339 (64.45) |  |
| Yes | 271 (36.04) | 84 (37.17) | 187 (35.55) |  |
| Delayed extubation, n(%) |  |  |  | 0.421 |
| No | 510 (67.82) | 158 (69.91) | 352 (66.92) |  |
| Yes | 242 (32.18) | 68 (30.09) | 174 (33.08) |  |
| Sex, n(%) |  |  |  | 0.901 |
| Male | 430 (57.18) | 130 (57.52) | 300 (57.03) |  |
| Female | 322 (42.82) | 96 (42.48) | 226 (42.97) |  |
| Smoking status, n(%) |  |  |  | 0.298 |
| No | 411 (54.65) | 117 (51.77) | 294 (55.89) |  |
| Yes | 341 (45.35) | 109 (48.23) | 232 (44.11) |  |
| Alcohol intake, n(%) |  |  |  | 0.551 |
| No | 537 (71.41) | 158 (69.91) | 379 (72.05) |  |
| Yes | 215 (28.59) | 68 (30.09) | 147 (27.95) |  |
| Hypertension, n(%) |  |  |  | 0.054 |
| No | 373 (49.60) | 100 (44.25) | 273 (51.90) |  |
| Yes | 379 (50.40) | 126 (55.75) | 253 (48.10) |  |
| Hyperlipidemia, n(%) |  |  |  | 0.768 |
| No | 185 (24.60) | 54 (23.89) | 131 (24.90) |  |
| Yes | 567 (75.40) | 172 (76.11) | 395 (75.10) |  |
| COPD, n(%) |  |  |  | 0.023 |
| No | 721 (95.88) | 211 (93.36) | 510 (96.96) |  |
| Yes | 31 (4.12) | 15 (6.64) | 16 (3.04) |  |
| CVD, n(%) |  |  |  | 0.269 |
| No | 673 (89.49) | 198 (87.61) | 475 (90.30) |  |
| Yes | 79 (10.51) | 28 (12.39) | 51 (9.70) |  |
| Tumor T stage, n(%) |  |  |  | 0.091 |
| T1/T2 | 414 (55.05) | 135 (59.73) | 279 (53.04) |  |
| T3/T4 | 338 (44.95) | 91 (40.27) | 247 (46.96) |  |
| Extensive maxillomandibular resection, n(%) |  |  |  | 0.111 |
| No | 532 (70.74) | 169 (74.78) | 363 (69.01) |  |
| Yes | 220 (29.26) | 57 (25.22) | 163 (30.99) |  |
| Bilateral neck dissection, n(%) |  |  |  | 0.385 |
| No | 597 (79.39) | 175 (77.43) | 422 (80.23) |  |
| Yes | 155 (20.61) | 51 (22.57) | 104 (19.77) |  |

| Model | AUC (95% CI) | Accuracy | Precision | Sensitivity | Specificity | F1 Score | Kappa | Youden's J | NPV |
| --- | --- | --- | --- | --- | --- | --- | --- | --- | --- |
| Logistic | 0.803 (0.740-0.858) | 0.756 | 0.615 | 0.375 | 0.907 | 0.466 | 0.319 | 0.282 | 0.785 |
| Decision Tree | 0.720 (0.642-0.800) | 0.729 | 0.528 | 0.438 | 0.845 | 0.479 | 0.298 | 0.282 | 0.791 |
| Random Forest | 0.859 (0.808-0.905) | 0.773 | 0.607 | 0.578 | 0.851 | 0.592 | 0.435 | 0.429 | 0.835 |
| XGBoost | 0.858 (0.810-0.905) | 0.778 | 0.659 | 0.453 | 0.907 | 0.537 | 0.397 | 0.360 | 0.807 |
| LightGBM | 0.851 (0.795-0.902) | 0.782 | 0.623 | 0.594 | 0.857 | 0.608 | 0.457 | 0.451 | 0.841 |
| SVM | 0.788 (0.720-0.849) | 0.724 | 0.538 | 0.219 | 0.925 | 0.311 | 0.176 | 0.144 | 0.749 |
| ANN | 0.741 (0.664-0.807) | 0.747 | 0.684 | 0.203 | 0.963 | 0.313 | 0.210 | 0.166 | 0.752 |

**Table S3. Predictive performance of seven machine learning models in the validation sets**
